# A Transformer–LSTM Hybrid Detector for OFDM-IM Signal Detection

**DOI:** 10.3390/e28010102

**Published:** 2026-01-14

**Authors:** Leijun Wang, Zian Tong, Kuan Wang, Jinfa Xie, Xidong Peng, Bolong Li, Jiawen Li, Xianxian Zeng, Jin Zhan, Rongjun Chen

**Affiliations:** 1 School of Computer Science, Guangdong Polytechnic Normal University, Guangzhou 510665, China; wangleijun@gpnu.edu.cn (L.W.); wangkuan@stu.gpnu.edu.cn (K.W.); xiejinfa@stu.gpnu.edu.cn (J.X.); pengxidong@stu.gpnu.edu.cn (X.P.); libolong@stu.gpnu.edu.cn (B.L.); zengxianxian@gpnu.edu.cn (X.Z.); chenrongjun@gpnu.edu.cn (R.C.); 2 Sound and Vibration Division P&R Measurement Technology Co., Ltd., Zhuhai 519125, China; zian.tong@prmeasure.com

**Keywords:** deep learning, long short-term memory (LSTM), orthogonal frequency division multiplexing with index modulation (OFDM-IM), transformer

## Abstract

This paper addresses the signal detection problem in orthogonal frequency division multiplexing with index modulation (OFDM-IM) systems using deep learning (DL) techniques. In particular, a DL-based detector termed FullTrans-IM is proposed, which integrates the Transformer architecture with long short-term memory (LSTM) networks. Unlike conventional methods that treat signal detection as a classification task, the proposed approach reformulates it as a sequence prediction problem by exploiting the sequence modeling capability of the Transformer’s decoder rather than relying solely on the encoder. This formulation enables the detector to effectively learn channel characteristics and modulation patterns, thereby improving detection accuracy and robustness. Simulation results demonstrate that the proposed FullTrans-IM detector achieves superior bit error rate (BER) performance compared with conventional methods such as zero-forcing (ZF) and existing DL-based detectors under Rayleigh fading channels.

## 1. Introduction

Orthogonal frequency division multiplexing with index modulation (OFDM-IM) [1] has emerged as a promising alternative to traditional OFDM technology in multicarrier systems. In OFDM-IM, only a subset of subcarriers is active, and both the activated subcarriers and their indices can carry information bits. This enables higher reliability and energy efficiency compared to OFDM, since it does not require extra power or bandwidth to carry data bits through the indices of active subcarriers. Furthermore, OFDM-IM provides an attractive trade-off between spectral efficiency and reliability by adjusting the number of active subcarriers. Although many variants of OFDM-IM schemes have been proposed to expand the design space for flexible communication systems [2], challenges such as computational complexity and power consumption in practical deployments still require further investigation. To address detection complexity, solutions such as the maximum subcarrier power detection (MSPD) algorithm have been proposed in [3], although they require optimization for specific settings. While a theoretical analysis of multi-carrier index keying orthogonal frequency division multiplexing (MCIK-OFDM) is provided in [4], its real-world applicability is limited. To address computational complexity and interference in multiple-input multiple-output orthogonal frequency division multiplexing with index modulation (MIMO-OFDM-IM) systems, sequential Monte Carlo (SMC)-based detection has shown significant performance improvements [5].

On the other hand, deep learning (DL), which has revolutionized fields such as computer vision and speech recognition [6,7], is increasingly being applied in wireless communications. A deep neural network (DNN)-based autoencoder framework has shown significant potential in improving bit error rate (BER) performance and spectral efficiency in OFDM systems [8]. In [9], DeepIM, a deep learning-based detector, was introduced for OFDM-IM systems. It achieves efficient signal detection with low complexity and near-optimal performance. Additionally, a dual-mode detector utilizing a convolutional neural network (CNN) and a DNN was proposed in [10], thereby enhancing detection accuracy by separating the processes for index and carrier bits. Similarly, a two-stage dilated convolutional neural network (TS-DCNN) was developed in [11], which improves BER performance and efficiency through separate detection of index and carrier bits. Moreover, deep recurrent neural networks (DRNNs), which utilize long short-term memory (LSTM), have demonstrated the ability to reduce complexity while maintaining high detection accuracy [12].

It is notable that the Transformer [13] network structure, which exhibits superior performance in various fields, was introduced into OFDM-IM detection in [14]. The TransIM model in [14] combines the Transformer networks with traditional post-processing to enhance BER performance. In our previous work [15], we explored Transformer models for coded OFDM systems and demonstrated their capability in signal detection process. Although some researchers have attempted to utilize Transformers in the communication tasks, to the best of our knowledge, none of the existing works has explored the potential of applying the full Transformer model, in particular performing classification using only the Transformer encoder layer. To maximize the Transformer’s potential, we develop a network employing the Transformer decoder designed specifically for OFDM-IM systems. Thus, in this paper, we propose a novel DL-based detector, named FullTrans-IM, which reformulates the detection problem as a sequence prediction task, so that the Transformer decoder can be used naturally. For comparison, we also design a network using only the Transformer encoder, termed TransEnc-IM, for the OFDM-IM system. Simulation results indicate that FullTrans-IM significantly outperforms conventional zero-forcing (ZF) and TransEnc-IM detectors under Rayleigh fading channels. The main contributions of this paper can be summarized as follows:

- Hybrid Transformer–LSTM architecture: A new FullTrans-IM framework is developed by combining the Transformer’s self-attention mechanism with LSTM-based temporal modeling, enabling the detector to effectively capture both global and sequential dependencies within the received OFDM-IM signals.
- Sequence prediction-based detection: Unlike conventional encoder-only approaches that treat detection as a static classification problem, the proposed FullTrans-IM reformulates signal detection as a sequence prediction task using the Transformer’s decoder, thereby enhancing prediction accuracy and robustness.
- Performance superiority and efficiency: Simulation results demonstrate that the proposed FullTrans-IM achieves significantly better BER performance and improved robustness compared with existing detectors, while maintaining a favorable trade-off between accuracy and computational complexity.

The remainder of the paper is organized as follows. Section 2 introduces the OFDM-IM system model, including the index modulation scheme; Section 3 describes the proposed FullTrans-IM network architecture in detail, covering the preprocessing module, Transformer-based model design, loss function formulation, and the offline training and online deployment processes; Section 4 discusses the simulation setup and analyzes BER performance under various modulation schemes; Finally, Section 5 concludes the paper and discusses potential directions for future research.

## 2. System Description

**Figure 1:**
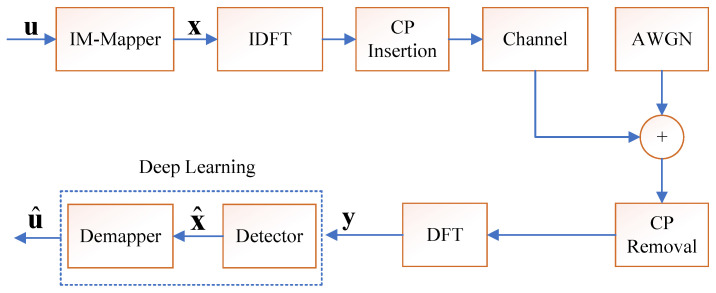
The block diagram of the OFDM-IM system.

In this paper, we consider a single-antenna OFDM-IM system over time-varying frequency-selective Rayleigh fading channels. The overall communication process of the OFDM-IM system is illustrated in Figure 1. Assuming that there are *N* subcarriers in the OFDM-IM system, an information bit sequence u is modulated into the frequency-domain transmitted OFDM symbol, denoted as $x={\left[x_{0},\ldots ,x_{N-1}\right]}^{T}$, where the notation ${\left[\cdot \right]}^{T}$ denotes the transpose of a vector. The time-domain symbols are generated by applying the inverse discrete Fourier transform (IDFT) to the frequency-domain data symbols. To eliminate inter-symbol interference (ISI) induced by channel dispersion, a cyclic prefix (CP) of length $N_{cp}\geq N_{tap}-1$ is appended to the beginning of each time-domain symbol. The resulting sequence is subsequently transmitted through a doubly selective channel characterized by $N_{tap}$ resolvable taps. At the receiver side, after the cyclic prefix is removed and the discrete Fourier transform (DFT) is performed, the received signal vector can be expressed as:

(1) $$y={\mathrm{FH}}_{t}F^{H}x+w=Hx+w,$$

where F denotes the unitary DFT matrix of dimension N×N, and ${\left(\cdot \right)}^{H}$ represents the Hermitian transpose of a matrix. The matrix $H_{t}$ represents the time-domain channel matrix, the detailed construction of which will be presented in the subsequent section. The matrix $H={\mathrm{FH}}_{t}F^{H}$ corresponds to the equivalent frequency-domain channel matrix. The vector w denotes additive white Gaussian noise (AWGN), which follows a complex normal distribution $\mathrm{CN}(0,\sigma^{2}I_{N})$.

### 2.1. The Channel Model

In an OFDM system, the discrete-time channel matrix elements are represented by the discrete-time impulse response $h_{n,m}$, where *n* denotes the subcarrier index, and *m* represents the tap index, with 0≤n≤N−1 and $0\leq m\leq N_{tap}-1$. According to Jakes’ Doppler spectrum model [16], the channel coefficient $h_{n,m}$ can be expressed as:

(2) $$h_{n,m}=\sqrt{\frac{P_{m}}{4M_{s}}}\left\{\sum_{i=1}^{M_{s}}\sqrt{2}e^{j\psi_{i}}\left[e^{j(\omega_{i}n+\phi )}+e^{-j(\omega_{i}n+\phi )}\right]\right\},$$

where $\omega_{i}=\omega_{d}\cos\alpha_{i}$, and the Doppler frequency parameters are defined as

$$\omega_{d}=\frac{2\pi \vartheta_{\max}}{N},\;\alpha_{i}=\frac{2\pi i-\pi +\theta }{4M_{s}},\;\;\;i=1,2,\ldots ,M_{s}.$$

In this work, $M_{s}$ denotes the number of sinusoids used to model the fading process, and is set to $M_{s}=32$. The term $P_{m}$ represents the power-delay profile (PDP), while $\vartheta_{max}$ corresponds to the normalized maximum Doppler shift. The random variables θ, ϕ and ψ are mutually independent and uniformly distributed over the interval [−π,π) for all *i*.

The time-domain channel matrix $H_{t}$ consists of nonzero elements generated according to Equation (2). Given the cyclic prefix and OFDM signal structure, $H_{t}$ can be decomposed as $H_{t}=L+U$, where L is an N×N lower-triangular matrix with nonzero elements $L_{n,m}=h_{n,n-m}$ for 0≤m≤n≤N−1. Similarly, U is an N×N upper-triangular matrix with nonzero elements $U_{n,m}=h_{n,N+n-m}$ for $0\leq n\leq N_{tap}-1,N-N_{tap}+1\leq m\leq N-1$, and n≤m.

### 2.2. The Index Modulation

In the OFDM-IM system, a block of OFDM containing *N* subcarriers is divided into *g* groups, with each group consisting of $N_{g}=\frac{N}{g}$ subcarriers. For each group, we select $n_{a}$ subcarriers to transmit the signals. The transmitted signal vector is denoted as $s_{\beta }=\left[s_{\beta ,0},\ldots ,s_{\beta ,n_{a}-1}\right]$, where β=0,…,g−1.

In the index modulation scheme, for any arbitrary group β, two constellations are involved. The first is the traditional two-dimensional *signal constellation* S⊂C with $\left|S\right|=2^{N_{s}}$, and we can define a one-to-one mapping $\varphi_{s}:F_{2}^{N_{s}}\to S$. Apparently, each transmitted symbol in $s_{\beta }$ is selected from S, i.e., $s_{\beta ,\gamma }\in S$, where $\gamma =0,\ldots ,n_{a}-1$. The second constellation, referred to as the *index constellation* I, determines the active subcarriers. The index constellation consists of $2^{N_{a}}$ Boolean vectors $I_{\beta }=\left(i_{\beta ,0},\ldots ,i_{\beta ,N_{g}-1}\right)\in {\left\{0,1\right\}}^{N_{g}}$, each with a Hamming weight of $n_{a}$, where $i_{\beta ,\gamma }=1$ indicates that the γ-th subcarrier is active. Let Na=log2Ngna, where ⌊x⌋ denotes the greatest integer not exceeding *x*. Then, we can define a one-to-one mapping $\varphi_{a}:F_{2}^{N_{a}}\to I$. With the above settings, we can further define a combined mapping $\varphi :F_{2}^{N_{a}+n_{a}N_{s}}\to X$ for each group in the OFDM-IM system.

As an example, consider an OFDM symbol with N=128 subcarriers divided into g=32 groups. Each group contains $N_{g}=4$ subcarriers, of which $n_{a}=2$ subcarriers are activated in each transmission. Consequently, there exist 42=6 possible index combinations, from which $2^{\left\lfloor \log_{2}6\right\rfloor }=4$ combinations are selected to form the index constellation. This selection guarantees that each subcarrier group maps an integer number of index bits. The index constellation can be defined as:

$$I=\left\{(1,1,0,0),(0,1,1,0),(0,0,1,1),(1,0,0,1)\right\}.$$

For each active subcarrier, *M*-ary quadrature amplitude modulation (QAM) or phase-shift keying (PSK) is implemented.

At the receiver, the ZF detection algorithm is used to mitigate inter-symbol interference or to preprocess signals for the deep learning-based detector. After processing with the pseudo-inverse matrix $H^{†}$, the received vector is expressed as:

(3) $$H^{†}y=x+H^{†}w.$$

where $H^{†}$ denotes the pseudo-inverse of the channel matrix H. For all possible index constellation I, the ZF detector estimates each nonzero element in x from the signal constellation S.

## 3. Proposed FullTrans-IM Detector

In this section, we first introduce the structure of the proposed FullTrans-IM network. Subsequently, we describe the offline training and online deployment processes of FullTrans-IM. In practical applications, although the FullTrans-IM detector can process multiple IM groups simultaneously, we illustrate its operation using a single group as an example.

### 3.1. Structure of FullTrans-IM Detector

**Figure 2:**
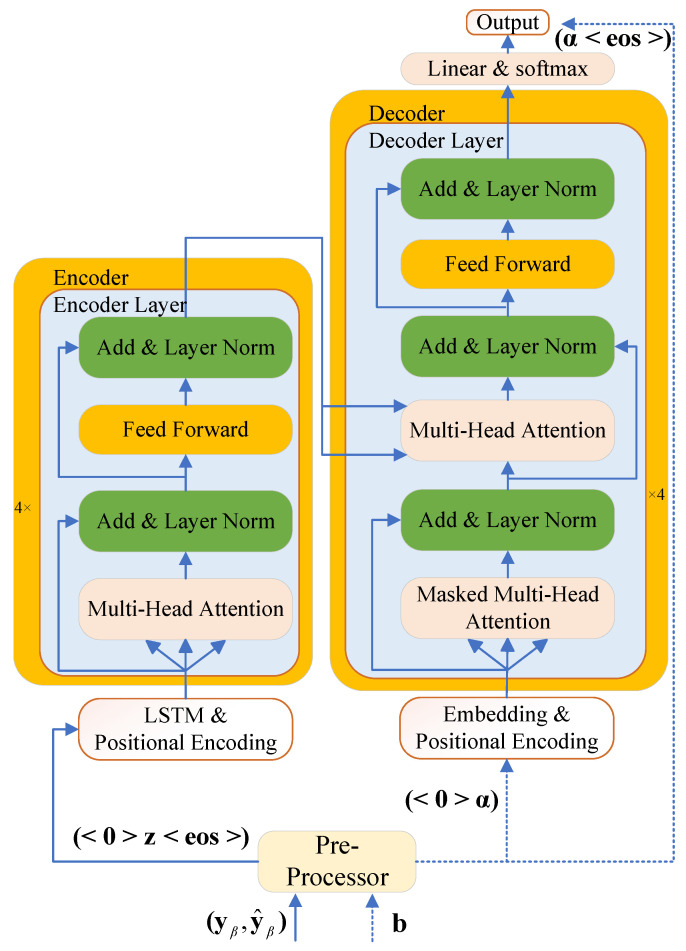
FullTrans-IM network structure.

As illustrated in Figure 2, the proposed FullTrans-IM network consists of two main components: a data preprocessor and a classical Transformer model. Specifically, the solid arrows in the architecture represent the data flow paths that are active during both the training and testing phases. In contrast, the dashed arrows denote paths that carry data only during the training phase and remain inactive during the online testing. The main modules of FullTrans-IM are introduced in detail below.

#### 3.1.1. Preprocessor

For an arbitrary group β, the preprocessor accepts two types of inputs. The first input type is the received signal vector before and after ZF equalization, denoted by $\left(y_{\beta },{\hat{y}}_{\beta }\right)$. These two vectors are extracted, respectively, from y in Equation (1) and $\hat{y}=H^{†}y$ in Equation (3) for a single group. The second input type is the bit information b corresponding to one group, which is used for training or evaluation. By concatenating the modulus of each element of $y_{\beta }^{⊤}$, the real part of ${\hat{y}}_{\beta }^{⊤}$, and the imaginary part of ${\hat{y}}_{\beta }^{⊤}$, we obtain the following row vector:

(4) $$z=\left[|y_{\beta }^{⊤}|\;ℜ\left\{{\hat{y}}_{\beta }^{⊤}\right\}\;ℑ\left\{{\hat{y}}_{\beta }^{⊤}\right\}\right].$$

In Equation (4), the symbol |·| denotes the element-wise modulus operation on a complex vector, whereas ℜ{·} and ℑ{·} represent the extraction of the real and imaginary parts, respectively. This representation, though mathematically redundant, provides multi-dimensional physical cues that enrich the network’s feature space. Specifically, the modulus component offers an energy-based feature that helps the model identify active subcarrier indices, whereas the real and imaginary components facilitate accurate reconstruction of the constellation symbols.

For the positional encoding of z, the tokens are first projected into a high-dimensional embedding space via a linear mapping layer. Subsequently, standard *sine* and *cosine* functions are applied to generate positional encodings, which are added to the embeddings to enable the Transformer to capture the sequential dependencies within the received signals.

For the bit sequence $b=(b_{0},b_{1},\ldots ,b_{n-1})$, a mapping function is defined as

(5) $$f\left(b_{i}\right)=\left\{\begin{array}{cc} i+1, & \mathrm{if}\;b_{i}=0,\\ i+n+1, & \mathrm{if}\;b_{i}=1, \end{array}\right.$$

where i=0,1,…,n−1, and $n=N_{a}+n_{a}N_{s}$ for an IM group. This mapping ensures that the values of $f(b_{i})$ fall within the range [1,2n], creating a distinct set of 2n tokens. Through this mapping, the bit sequence b is transformed into a real-valued vector $\alpha =\left[f\left(b_{0}\right),f\left(b_{1}\right),\ldots ,f\left(b_{n-1}\right)\right]$. To align with the sequence-to-sequence processing format of the Transformer model, both the input z and the output vector α are augmented with special functional tokens. Specifically, the sequence is prepended with a start token 〈0〉 (defined as 0 to trigger the start of decoding) and appended with an end token 〈eos〉, where 〈eos〉 is defined as 2n+1.

For example, consider the bit sequence b=(0,1,0,1,1,0). When n=6, the corresponding mappings, see Equation (5), are obtained as α=(1,8,3,10,11,6) and 〈eos〉=13.

#### 3.1.2. Transformer Model

As illustrated in Figure 2, a modified Transformer architecture is employed in the FullTrans-IM network. Both the encoder and decoder consist of four stacked layers, each with an embedding dimension of 128. Each layer contains a self-attention module followed by a feed-forward network with a hidden dimension of 512. The selection of a four-layer architecture was determined based on empirical evaluation. Experimental results showed that increasing the number of layers did not lead to noticeable improvements in performance. Instead, deeper architectures tended to cause overfitting and significantly increased computational complexity. In contrast, using fewer layers reduced the model’s ability to represent and learn the complex temporal and spectral characteristics of time-varying frequency-selective Rayleigh fading channels. Therefore, a four-layer configuration was adopted as the optimal balance between model expressiveness, generalization capability, and computational efficiency.

In both the encoder and decoder, the input tokens are embedded into 128-dimensional dense vectors. The encoder further processes these embeddings using a two-layer LSTM with an input dimension of 1 and a hidden state dimension of 128, capturing temporal dependencies in the sequence before passing the resulting representations through the encoder stack. In contrast, the decoder directly feeds the embeddings into the decoder stack, which incorporates self-attention and cross-attention mechanisms to integrate the encoded features with the decoder’s current state. Finally, layer normalization is applied at the end of both the encoder and decoder stacks before generating the final output.

#### 3.1.3. Multi-Head Attention

The multi-head attention mechanism relies on the query (Q), key (K), and value (V) [13], which are used to compute attention weights. These weights determine the relevance of different elements in the input sequence to each position in the output representation. The multi-head attention operation is defined as:

$$\mathrm{MultiHead}\left(Q,K,V\right)=\mathrm{Concat}\left({\mathrm{head}}_{1},\ldots ,{\mathrm{head}}_{h}\right)W^{O},$$

where each attention head is computed as

$${\mathrm{head}}_{i}=\mathrm{Attention}\left({\mathrm{QW}}_{i}^{Q},{\mathrm{KW}}_{i}^{K},{\mathrm{VW}}_{i}^{V}\right).$$

Here, $W_{i}^{Q}$, $W_{i}^{K}$, and $W_{i}^{V}$ denote the learned projection matrices for the queries, keys, and values in each head, respectively. The concatenated outputs from all heads are then linearly transformed by the output projection matrix $W^{O}$. The scaled dot-product attention mechanism within each head is expressed as:

$$\mathrm{Attention}\left(Q,K,V\right)=\mathrm{softmax}\left(\frac{{\mathrm{QK}}^{⊤}}{\sqrt{d_{k}}}\right)V,$$

where $d_{k}$ denotes the dimensionality of the key vectors. The scaling factor $\sqrt{d_{k}}$ prevents the dot-product values from becoming excessively large, thereby ensuring numerical stability during the softmax computation.

#### 3.1.4. Loss Function

In this paper, the cross-entropy loss is employed to measure the divergence between the true label distribution and the predicted probability distribution generated by the model. By minimizing this loss, the model learns to generate output probabilities that more closely align with the true labels. The loss function is defined as

$$L=-\frac{1}{M^{\prime }n}\sum_{i=0}^{M^{\prime }-1}\sum_{j=0}^{n-1}\log\left(\frac{\exp(\theta_{i,\varphi (j)})}{\sum_{k=0}^{2n+1}\exp\left(\theta_{i,k}\right)}\right),$$

where $M^{\prime }$ denotes the total number of samples in the dataset, and *n* represents the number of bits corresponding to one IM group. Furthermore, $\theta_{i,k}$ denotes the logit corresponding to the *k*-th class of the *i*-th sample. Similarly, $\theta_{i,\varphi (j)}$ represents the logit associated with the correct class of the *j*-th bit.

### 3.2. Offline Training and Online Deployment

**Figure 3:**
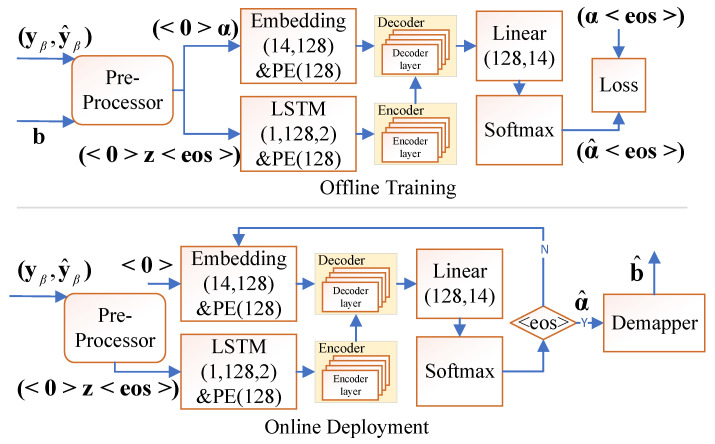
Offline Training and Online Deployment Framework.

The overall framework of offline training and online deployment is illustrated in Figure 3. During the offline training phase, the model is trained to learn the relationships among the input data $\left(y_{\beta },{\hat{y}}_{\beta }\right)$, the mapped vector α derived from b, and the corresponding output sequences $\hat{\alpha }$. The data $\left(y_{\beta },{\hat{y}}_{\beta }\right)$ are pre-processed into z, which is then fed into the encoder for feature extraction. The decoder then predicts the output sequence $\hat{\alpha }$ based on the features extracted by the encoder. Finally, the predicted sequence is compared with α, and the cross-entropy loss is computed to optimize the model parameters.

In the online deployment phase, only the data $\left(y_{\beta },{\hat{y}}_{\beta }\right)$ are passed through the encoder, which extracts the relevant features. The decoder begins with the start token 〈0〉 and generates the output sequence sequentially, relying on both the encoder features and its own previously generated outputs until the end token 〈eos〉 is produced. Finally, the predicted bit sequence $\hat{b}$ is reconstructed from the generated sequence $\hat{\alpha }$.

## 4. Simulation Results

In this section, the proposed FullTrans-IM and TransEnc-IM models are evaluated, where TransEnc-IM denotes a standard Transformer architecture that utilizes only the encoder layer. Additionally, the traditional ZF detector and a DNN-IM detector are included for comparison. The simulation results demonstrate and compare the performance of the proposed and benchmark methods under various modulation schemes.

### 4.1. BER Performance

The BER is evaluated via Monte Carlo simulations. For each signal-to-noise ratio (SNR) point, the predicted bit sequence $\hat{b}$ produced by the FullTrans-IM detector and demapper is compared with the original transmitted bit sequence b on a bit-by-bit basis. For the dataset, a 9:1 split is applied between the training and testing sets, ensuring that the model is trained on diverse channel conditions while maintaining sufficient unseen data for performance evaluation.

The experiments are conducted based on three key parameters, namely $\left(N_{g},n_{a},M\right)$, where $N_{g}$ denotes the number of subcarriers within each group; $n_{a}$, which represents the number of active subcarriers selected for transmission; and *M*, which denotes the modulation order. In the following simulations, an OFDM symbol with N=64 subcarriers is utilized. The OFDM symbol is divided into g=16 groups, each containing $N_{g}=4$ subcarriers, where $n_{a}=2$ active subcarriers are selected per group. Moreover, we employ the index constellation $I=\left\{(1,1,0,0),(0,1,1,0),(0,0,1,1),(1,0,0,1)\right\}$. Additionally, M=4 corresponds to QPSK modulation, whereas, M=8 corresponds to 8-PSK modulation. To ensure the fairness and consistency of the performance comparison among different detection methods, all simulations are conducted under the same experimental configuration, as summarized in Table 1.

**Figure 4:**
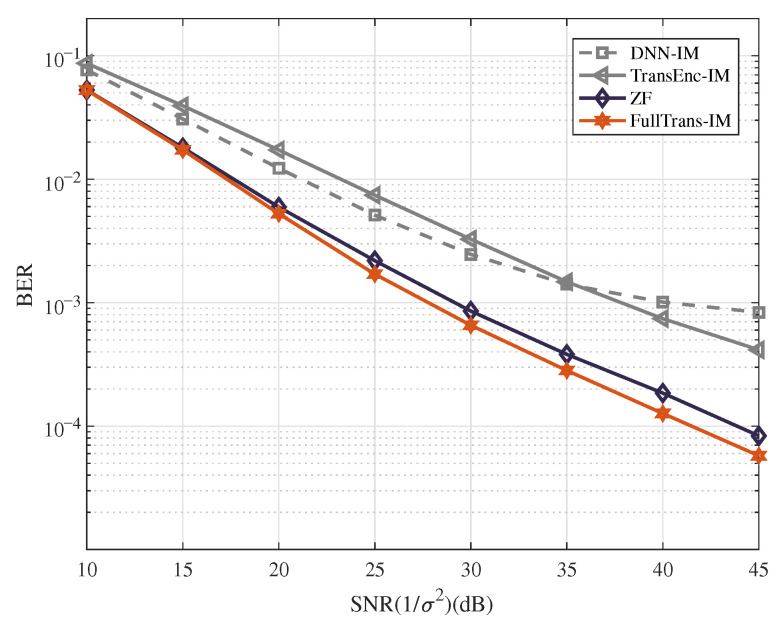
BER performance of the OFDM-IM system with the different types of detector and $\left(N_{g},n_{a},M\right)=\left(4,2,4\right)$.

**Example** **1.**  *In this example, the BER performance is evaluated under various SNR values with parameter settings of $\left(N_{g},n_{a},M\right)=\left(4,2,4\right)$. The training dataset comprises mixed SNR values ranging from 1 dB to 30 dB, with each SNR level utilizing 9,662,000 bits. The BER performance comparison is illustrated in Figure 4. From the figure, we have the following observations.*

- *The OFDM-IM system employing the FullTrans-IM detector achieves superior BER performance compared with the ZF, TransEnc-IM, and DNN-IM detectors.*
- *Both the TransEnc-IM and DNN-IM detectors exhibit an error floor in the high-SNR region, indicating limited generalization capability under low-noise conditions.*
- *At a BER of $10^{-4}$, the FullTrans-IM detector achieves approximately a 2.5 dB gain over the ZF detector.*

**Figure 5:**
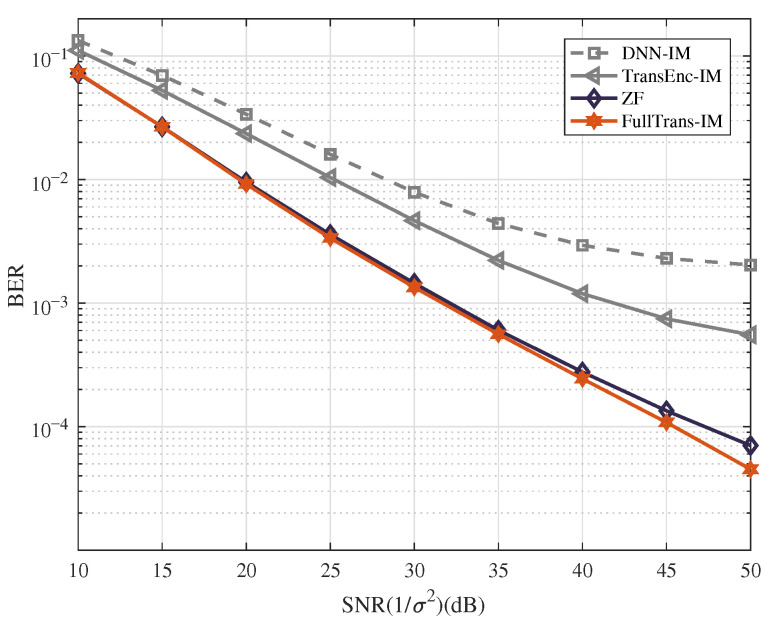
BER performance of the OFDM-IM system with the different types of detector and $\left(N_{g},n_{a},M\right)=\left(4,2,8\right)$.

**Example** **2.**  *In this example, the BER performance is evaluated under various SNR values with parameter settings of $\left(N_{g},n_{a},M\right)=\left(4,2,8\right)$. The training dataset is similarly generated using mixed SNR values ranging from 1 dB to 30 dB, with 12,882,000 bits employed per SNR level. The BER performance comparison is illustrated in Figure 5. From the figure, we have the following observations.*

- *The BER performance results follow a consistent trend with those observed in Example 1. The FullTrans-IM detector again achieves the best overall BER performance among all compared schemes.*
- *At a BER of $10^{-4}$, the FullTrans-IM detector attains approximately a 2 dB gain over the ZF detector.*

### 4.2. Training Loss Performance

**Figure 6:**
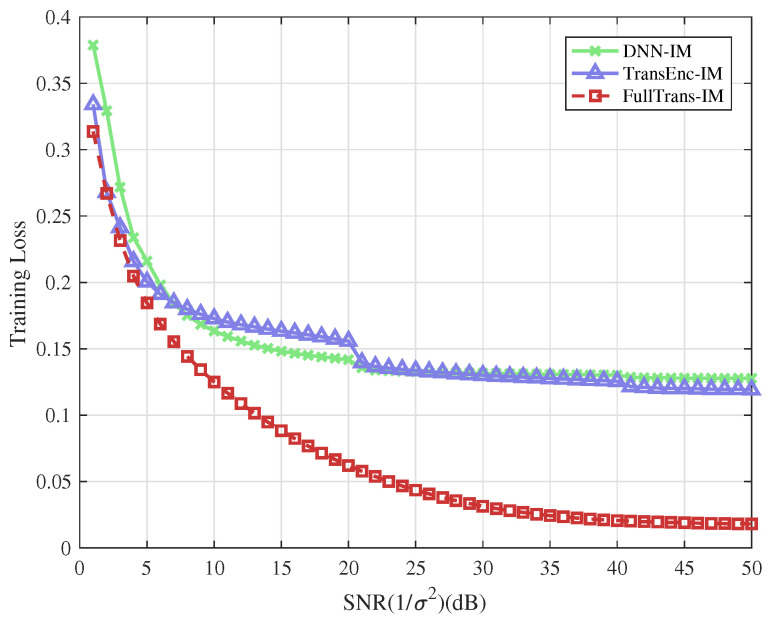
Training loss performance of the different types of deep learning based detector for OFDM-IM systems and $\left(N_{g},n_{a},M\right)=\left(4,2,4\right)$.

**Figure 7:**
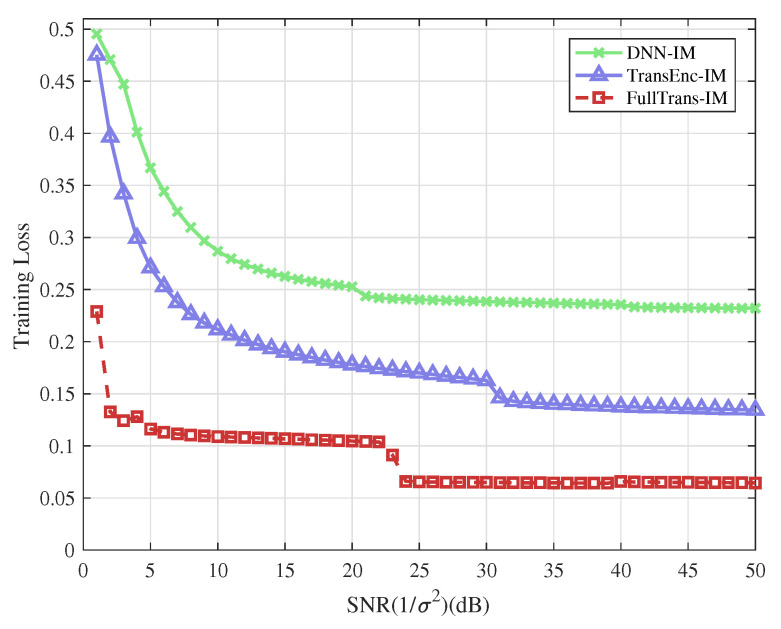
Training loss performance of the different types of deep learning based detector for OFDM-IM systems and $\left(N_{g},n_{a},M\right)=\left(4,2,8\right)$.

Corresponding to the schemes discussed in the examples above, the training losses of different neural network-based detectors are presented for comparison. The training losses of FullTrans-IM, TransEnc-IM, and DNN-IM are shown in Figure 6 and Figure 7, corresponding to the cases where $\left(N_{g},n_{a},M\right)$ equals (4,2,4) and (4,2,8), respectively. As shown in the figures, the FullTrans-IM consistently achieves lower training loss compared with both TransEnc-IM and DNN-IM, particularly in the high-SNR region. When $\left(N_{g},n_{a},M\right)=\left(4,2,8\right)$, the TransEnc-IM demonstrates superior performance compared with DNN-IM, indicating that attention-based architectures remain more stable than conventional feed-forward designs as modulation complexity increases. The FullTrans-IM, however, exhibits the most rapid reduction in training loss and converges to a substantially lower steady-state value, demonstrating its enhanced optimization capability and stronger representational power.

It is observed that the training loss curves of the 6-bit and 8-bit OFDM-IM schemes exhibit distinct convergence behaviors. In particular, a noticeable loss drop occurs at a certain training stage, which is more pronounced in the 8-bit case. This phenomenon is mainly attributed to the substantially increased combinatorial detection complexity introduced by the higher bit mapping in the 8-bit OFDM-IM scheme. During the early training stage, the model experiences difficulty in jointly learning the coupling between subcarrier index selection and symbol modulation, resulting in a relatively slow reduction of the training loss. As training proceeds, once the underlying structural characteristics of OFDM-IM are effectively captured, a large proportion of training samples can be correctly decoded. Consequently, the training loss exhibits a sudden decrease, indicating that the model has transitioned from coarse representation learning to structured detection.

Compared with the 6-bit scheme, the 8-bit OFDM-IM configuration involves a larger joint search space and stronger index-symbol dependency, which delays the formation of reliable internal representations and leads to a more evident loss drop. These results suggest that the proposed model is capable of learning the intrinsic structure of OFDM-IM signals, especially under higher mapping complexity, thereby demonstrating its effectiveness in handling challenging index modulation detection tasks.

These observations highlight that the FullTrans-IM effectively leverages its joint encoder–decoder structure to capture both local feature dependencies and long-term sequential correlations within OFDM-IM signals. As a result, it achieves more efficient gradient propagation and improved convergence stability during training. This also implies that the FullTrans-IM model generalizes more effectively across varying SNR conditions, which contributes to its superior detection accuracy in the test phase.

Overall, these results confirm that integrating both encoder and decoder components within the FullTrans-IM architecture enables superior feature extraction and optimization stability compared with conventional Transformer or DNN-based structures, making it a robust and scalable solution for OFDM-IM detection.

### 4.3. Complexity Comparison

In Table 2, we provide the time complexity of various detection methods, including FullTrans-IM, TransEnc-IM, DNN-IM and ZF, with different modulation schemes. The evaluation is conducted using a dataset containing $10^{5}$ bits, and the processing time is measured in seconds (s). To ensure a fair comparison with the traditional ZF algorithm, the neural network models (originally trained in PyTorch) were converted into a C++ compatible format via LibTorch. All detection schemes were then executed and timed within a unified Visual Studio 2022 environment on the hardware platform described in Section 4.1.

As shown in Table 2, although the FullTrans-IM method exhibits higher time complexity compared to TransEnc-IM and DNN-IM, with times of 0.835 s when $\left(N_{g},n_{a},M\right)=\left(4,2,4\right)$ and 0.899 s when $\left(N_{g},n_{a},M\right)=\left(4,2,8\right)$, it still demonstrates a significant advantage over the ZF detector. The ZF detector has a time complexity of 9.097 s when $\left(N_{g},n_{a},M\right)=\left(4,2,4\right)$ and 10.523 s when $\left(N_{g},n_{a},M\right)=\left(4,2,8\right)$, which is more than an order of magnitude higher than that of FullTrans-IM.

Furthermore, this reduction in processing time is directly linked to the energy efficiency of the detection process. In practical wireless communication systems, energy consumption is proportional to the active execution time of the processor. Since the FullTrans-IM detector significantly shortens the inference latency compared to the traditional ZF method, it effectively reduces the energy required per bit for signal recovery.

These results indicate that although the full Transformer architecture introduces additional computational overhead, the FullTrans-IM detector achieves an excellent trade-off between accuracy and efficiency. Its superior processing speed relative to ZF highlights the effectiveness of Transformer-based sequence modeling for OFDM-IM detection while maintaining practical computational feasibility.

## 5. Conclusions

In this paper, a novel FullTrans-IM detector has been proposed for OFDM-IM systems by integrating the Transformer architecture with LSTM networks. In the proposed detector, signal detection is formulated as a sequence prediction problem, leveraging the powerful sequence modeling capability of the Transformer’s decoder rather than treating detection purely as a classification task based on the encoder. Simulation results demonstrate that the FullTrans-IM detector achieves superior BER performance and enhanced robustness compared with the TransEnc-IM, DNN-IM, and ZF detectors under Rayleigh fading channels. Furthermore, the proposed method attains a good trade-off between detection accuracy and computational complexity, highlighting its potential for practical deployment in next-generation wireless communication systems. Future work may explore extending the FullTrans-IM framework to MIMO and higher-order modulation scenarios to further enhance detection efficiency and scalability.

## Figures and Tables

**Table 1:** Simulation and experimental configuration.

Configuration Item	Value
Computing Platform	Desktop computer (MSI)
Processor	Intel i7-11700 @ 2.5 GHz
Memory	64 GB
Graphics Card	NVIDIA RTX 3090/24 GB
Programming Language	Python 3.10
Deep Learning Framework	PyTorch 1.12.0 (CUDA 11.3)
Model Parameters	Learning rate = 0.0003, Epochs = 50
Channel Model	Rayleigh fading channel
Performance Metric	BER

**Table 2:** Comparison of time complexity of the detectors.

$\left(N_{g},n_{a},M\right)$	DNN-IM	TransEnc-IM	FullTrans-IM	ZF
(4, 2, 4)	0.031 s	0.324 s	0.835 s	9.097 s
(4, 2, 8)	0.041 s	0.473 s	0.899 s	10.523 s

## Data Availability

The data presented in this study are available on request from the corresponding authors.

## Abbreviations

The following abbreviations are used in this manuscript:

AWGN	additive white Gaussian noise
BER	bit error rate
CNN	convolutional neural network
CP	cyclic prefix
DFT	discrete Fourier transform
DL	deep learning
DNN	deep neural network
DRNNs	deep recurrent neural networks
IDFT	inverse discrete Fourier transform
IM	index modulation
ISI	inter-symbol interference
LSTM	long short-term memory
MCIK-OFDM	multi-carrier index keying orthogonal frequency division multiplexing
MIMO	multiple-input multiple-output
MIMO-OFDM-IM	MIMO orthogonal frequency division multiplexing with index modulation
MSPD	maximum subcarrier power detection
OFDM	orthogonal frequency division multiplexing
OFDM-IM	OFDM with index modulation
PDP	power-delay profile
PSK	phase-shift keying
QAM	quadrature amplitude modulation
SMC	sequential Monte Carlo
SNR	signal-to-noise ratio
TS-DCNN	two-stage dilated convolutional neural network
ZF	zero forcing
